# Drug therapy problems for patients with tuberculosis and HIV/AIDS at a reference hospital

**DOI:** 10.31744/einstein_journal/2019AO4696

**Published:** 2019-08-19

**Authors:** Natália Helena de Resende, Silvana Spíndola de Miranda, Maria das Graças Braga Ceccato, João Paulo Amaral Haddad, Adriano Max Moreira Reis, Dirce Inês da Silva, Wânia da Silva Carvalho

**Affiliations:** 1 Universidade Federal de Minas Gerais, Belo Horizonte, MG, Brazil; 2 Fundação Hospitalar do Estado de Minas Gerais, Hospital Eduardo de Menezes, Belo Horizonte, MG, Brasil

**Keywords:** HIV, Acquired immunodeficiency syndrome, Tuberculosis, Pharmaceutical preparations

## Abstract

**Objective:**

To determine the frequency of drug therapy problem in the treatment of patients with tuberculosis and HIV/AIDS.

**Methods:**

Data were obtained through a cross-sectional study conducted between September 2015 and December 2016 at a reference hospital in infectious diseases in Belo Horizonte (MG), Brazil. Sociodemographic, clinical, behavioral and pharmacotherapeutic variables were evaluated through a semi-structured questionnaire. Drug-related problems of pharmaceutical care were classified using the Pharmacotherapy Workup method. Factors associated with indication, effectiveness, safety and compliance drug therapy problem were assessed through multiple logistic regression.

**Results:**

We evaluated 81 patients, and 80% presented at least one drug therapy problem, with indication and adherence drug therapy problem being the most frequent. The factors associated with drug therapy problem were age, marital status, new case, ethnicity, time of HIV diagnosis and time to treat tuberculosis.

**Conclusion:**

The frequency of drug therapy problem in coinfected patients was high and the identification of the main drug therapy problem and associated factors may lead the multiprofessional health team to ensure the use of the most indicated, effective, safe and convenient medicines for the patients clinical condition. Tuberculosis and HIV/AIDS coinfected individuals aged over 40 years are more likely to have drug therapy problems during treatment; in that, the most frequente are those that signal toward need of medication for an untreated health condition and non-compliance to treatment. Thus, older patients, unmarried or married, who have treated tuberculosis before, with a shorter time to tuberculosis treatment and longer time to diagnose HIV/AIDS, should receive special attention and be better followed by a multiprofessional health team because they indicate a higher chance of presenting Problems related to the use of non-adherent drugs.

## INTRODUCTION

The human immunodeficiency virus (HIV) infection represents a major challenge to tuberculosis (TB) control worldwide. The greatest susceptibility for developing TB in patients with HIV/AIDS is explained by the immune response to *Mycobacterium tuberculosis*. As a consequence, there is fast progression of the HIV.^(^^1^^)^

In 2016, there were an estimated 10.6 million new cases of TB in the world, and 10% (1.1 million) of them were also infected by HIV. The TB/HIV-AIDS coinfection accounts for the increase in mortality rates worldwide. In 2016, there were an estimated 1.5 million deaths due to TB in HIV negative patients and an additional 374 thousand patients who died due to coinfection.^(^^2^^)^ Combined treatment of TB and HIV/AIDS decreases mortality, TB relapse, and transmission of both diseases in the community.^(^^3^^)^ For new cases of TB, treatment is based on the combination of four drugs, rifampin, isoniazid, pyrazinamide and ethambutol, during the intensive phase, lasting two months. Rifampin and isoniazid are used for 4 months in the maintenance phase.^(^^4^^)^ In Brazil, the recommended initial AIDS therapy during the study period was the combination of three antiretroviral drugs, two nucleoside reverse transcriptase inhibitors (NRTI) associated with a non-nucleoside reverse transcriptase inhibitor (NNRTI). The first line regimen comprised tenofovir (TDF), lamivudine (3TC) and efavirenz (EFV). The presentation of this regimen is a combined fixed dose of the three drugs. Second line treatment is recommended in scenarios in which EFV and nevirapine cannot be used; in these cases, they are replaced by a protease inhibitor (PI).^(^^5^^)^

Simultaneous treatment of both infections, with any drugs, predisposes to the occurrence of adverse reactions, drug interactions and the possibility of compromising treatment compliance.^(^^6^^,^^7^^)^ In this scenario, a multiprofessional approach is essential for these patients in order to prevent, identify and solve drug therapy problems (DTP), that is, any undesirable event experienced by the patient that involves drug treatment and interferes in attaining desired therapeutic objectives.^(^^8^^)^

## OBJECTIVE

To determine the frequency of drug therapy problems associated with treatment indication, effectiveness, safety and compliance of patients with tuberculosis and HIV/AIDS, in addition to assess associated factors.

## METHODS

A cross-sectional analytical study, carried out between September 2015 and December 2016, at *Hospital Eduardo de Menezes* (HEM) of the *Fundação Hospitalar do Estado de Minas Gerais* (FHEMIG) in the city of Belo Horizonte (State of Minas Gerais - MG). The hospital is a reference for TB, HIV/AIDS and other infectious diseases.

The study was approved by the Research Ethics Committee of the *Universidade Federal de Minas Gerais,* no. 3434458, CAAE: 23692713.30000.5149 and of FHEMIG, no. 696.759, CAAE: 23692713.2.3001.5124.

The study included patients 18 years of age and over, with diagnosis of TB and HIV/AIDS, who began treatment of TB starting September 2015 and concurrent to the diagnosis of HIV/AIDS.

Patients coinfected with TB and HIV/AIDS that had a cognition *deficit*, or treatment abandonment, death, transfer to another health service or change of diagnosis previous to approach, were excluded.

Calculation of sample size took into account a sample error of 10%, 95% level of confidence, 50% frequency of DTP and the population of coinfected individuals seen at the hospital in 2014, which totaled up 136 patients. A sample of 57 was calculated and, considering a likely refusal rate of 30%, the minimum sample required was 74 patients.

Patients on anti-TB medication were identified by prescriptions and pharmacy service drug dispensation spreadsheets of the organizations included in the study. Afterwards, HIV diagnosis was checked on medical charts, FHEMIG Integrated Hospital Management System and on the Medication Logistic System (SICLOM) of the Sexually Transmitted Infections/AIDS and Viral Hepatitis Program (http://azt.aids.gov.br/) of the Ministry of Health. Data were collected by patient interviews using a semi-structured questionnaire at the HEM outpatient clinic, ward, and day-hospital.

Explanatory variables studied were grouped into sociodemographic and economic characteristics (sex, age, living alone, place of residence, marital status, schooling level, ethnicity, income and occupation); behavioral characteristics (smoking, use of alcohol and illicit drugs); clinical characteristics (clinical form of TB, time since HIV/AIDS diagnosis, time on TB treatment, hospitalization on date of interview, new case, associated diseases, viral load and CD4 lymphocyte T); and drug therapy-related characteristics (treatment regimens for TB and HIV/AIDS, number of medications used).

Response variables were the DTP, studied using a questionnaire adapted from Cipolle et al., and classified by the Pharmacotherapy Workup method, described in table 1.^(^^9^^)^

**Table 1:** Parameters for classification of each drug-related problem

Parameter	Classification of DRP
Need	DRP 1 – Unnecessary medication
	A - There is no indication
	B - Dual therapy
	C - Indicated non-drug therapy
	D - Predictable and preventable adverse drug reaction
	E- Recreational use of drugs
	DRP 2 – Needs additional pharmacotherapy
	A - Untreated condition
	B - Preventive – prophylactic
	C - Synergism – augmentation
Effectiveness	DRP 3 – Needs a different medication
	A - Most effective medication available
	B - Condition refractory to the medication
	C - Inappropriate dosage form
	D - Not effective for the condition
	DRP 4 – Very low dose
	A - Wrong dose
	B - Inappropriate intervals
	C - Drug interaction
	D - Inappropriate duration
Safety	DRP 5 – Adverse reaction to the drug
	A - Undesirable effect
	B - Unsafe medication for the patient
	C - Very fast administration
	D - Allergic reaction
	E - It is contraindicated
	DRP 6 – Very high dose
	A - Wrong dose
	B - Inappropriate intervals
	C- Inappropriate duration
	D - Drug interaction
	E - Incorrect administration
Compliance	DRP 7 – Does not follow the instructions
	A - Does not understand the instructions
	B - Patient prefers not to take the medication
	C - Patient forgets to take medication
	D - Very expensive product
	E - Patient not able to swallow or administer medication
	F - Product not available

Source: Adapted from Cipolle RJ, Strand LM, Morley PC. Pharmaceutical care practice: the clinician's guide. 2nd ed. New York: McGraw-Hill. 2004. 394 p.

DRP: drug-related problem.

### Statistical analysis

Data obtained were initially submitted to a descriptive analysis, including the description of the population studied, and distributions of frequencies of categorical variables and central tendency measurements, such as mean, median and standard deviation, for quantitative variables.

Descriptive analysis included the determination of the proportion of DTP according to recommended classifications.

The association among all characteristics selected (sociodemographic, behavioral, clinical data and pharmacotherapeutic profile) and indication, effectiveness, safety and compliance DTP was assessed using multiple logistic regression.

Independent variables with a p<0.20 were included in the multivariate model. The Hosmer-Lemeshow model adjustment test was performed. Data were analyzed using Stata version 12.0 program (Stata Corporation, College Station, USA).

Variables with a p<0.05 remained in the final model.

## RESULTS

**Figure 1:**
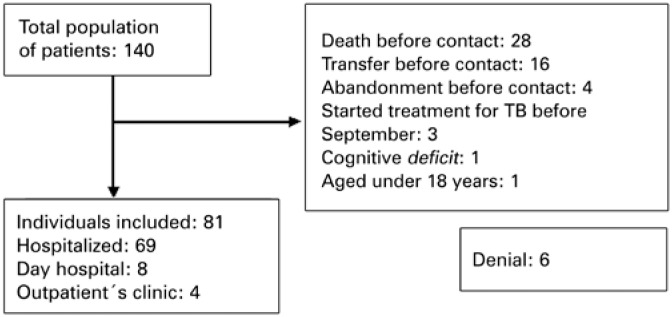
Population of patients coinfected with tuberculosis and HIV/AIDS to include in the study

Of the 140 coinfected patients during the study period, 59 patients were excluded for different reasons (Figure 1). The final sample for analysis comprised 81 patients.

Table 2 shows that 62 patients (77%) were men and that the mean age was 40 years. Among the 81 coinfected patients, 63 were single (78%), 67 declared themselves brown or black (83%) and 59 had up to 8 years of schooling (73%). Most had income (86%) and an occupation (72%).

**Table 2:** Sociodemographic, behavioral, clinical and pharmacotherapeutic characteristics of patients coinfected with tuberculosis and HIV/AIDS

Variables		n (%)
Sociodemographic		
	Male	62 (77)
	>40 years	39 (52)
	Single	63 (78)
	Up to 8 years of schooling	59 (73)
	Black or brown skin	67 (83)
	Income	70 (86)
Behavioral		
	Smoker	33 (41)
	Alcohol abuse	39 (48)
	Drugs	18 (22)
Clínical		
	Pulmonary TB	46 (57)
	ExtrapulmonaryTB	27 (33)
	Pulmonary + extrapulmonary TB	8 (10)
	Associated diseases	62 (77)
	Up to 1 year since HIV diagnosis	44 (54)
	Up to 2 months of TB treatment	71 (88)
	Undetectable viral load	10 (12)
	CD4 T Lymphocyte <200 cells/µL	59 (73)
Pharmacotherapeutical		
	First line TB treatment regimen	69 (85)
	No ART regimen	40 (49)
	First line ART	35 (43)
	Second line ART	6 (7)
Number of drugs		
	<10	12 (15)
	≥10	69 (85)

TB: tuberculosis; ART: antirretroviral therapy.

The most frequent clinical form of TB was pulmonary (57%), followed by extrapulmonary (33%). Of the extrapulmonary presentations, meningo-encephalic TB was the most frequent, representing 33% of patients, followed by ganglionary (26%) and miliary (26%). Of the 81 patients, 62 (77%) had other associated conditions, in addition to coinfection, such as candidiasis (23%), pneumonia (12%) and cytomegalovirus infection (10%). Moreover, alcohol abuse was observed in 39 patients (48%), smoking in 33 (41%) and use of illicit drugs in 18 (22%). Regarding variables related to drug treatment, 69 used first-line regimen comprised of rifampin, isoniazid, pyrazinamide and ethambutol for TB (85%), and 40 (49%) were not on any antiretroviral regimen.

Some patients were on a special regimen to treat TB due to adverse reactions already being treated at the time of data collection.

At the time of the interview, 71 patients (88%) had been treating TB for up to 2 months. The diagnosis of HIV occurred up to 1 year before the date of the interview in 44 patients (54%). Most patients that were not on antiretroviral therapy were on the intensive phase treatment of TB (95%).

Of the 81 patients interviewed, 65 (80.0%) had at least one DTP, and 46 patients (57%) had additional requirement DTP, 39 (48%) treatment compliance, 5 (6%) effectiveness, and 3 (4%) safety DTP.

We identified 110 DTP, 57 of which (52%) were related to requiring additional pharmacotherapy. Of these, for 20 (35%) it was due to a non-treated condition and for 37 (65%) to requiring prophylaxis. Pyridoxine supplementation due to risk of peripheral neuropathy was classified as prophylaxis requirement DTP.

There were 39 cases of non-adherence to treatment DTP (35%). Causes of non-adherence were: preferring not to take medication (53.8%), forgetting to take medication (33%), medication not available (8%) and not understanding instructions (5%).

In the univariate analysis of categorical variables, according to presence or absence of any type of DTP, the only variable presenting significance was age (p=0.046); in that, the likelihood of presenting a DTP by an individual aged over 40 years was 3.5 greater (95% confidence interval − 95%CI: 1.020-12.000).

Tables 3 to 6 present the results of the univariate and multivariate analyses with characteristics associated with indication, effectiveness, safety and compliance DTP, respectively.

**Table 3:** Factors associated with drug therapy problems - indication

Variable			Univariate analysis		Multivariate analysis	
			Odds ratio (95%CI)	p value	Odds ratio (95%CI)	p value
Sociodemographic						
	Sex					
		Male	1.246 (0.444-3.498)	0.676		
		Female	1.00			
	Age					
		>40 years	1.786 (0.733-4.353)	0.20		
		≤40 years	1.00			
	Ethnicity					
		Black or brown	0.465 (0.132-1.630)	0.23		
		White	1.00			
	Place of residence					
		Metropolitan region	1.379 (0.404-0.711)	0.61		
		Other municipalities	1.00			
	Marital status					
		Single or widowed	0.525 (0.177-1.560)	0.25		
		Married or common law union				
	Occupation					
		Yes	0.612 (0.225-1.667)	0.34		
		No	1.00			
Behavioral						
	Smoker					
		No	0.995 (0.404-2.448)	0.99		
		Yes	1.00			
	Alcohol					
		Yes	0.607 (0.249-1.484)	0.27		
		No	1.00			
Clinical						
	Type of TB diagnosis					
		Likely	0.690 (0.284-1.665)	0.41		
		Confirmed	1.00			
	Time since HIV/AIDS diagnosis					
		Up to 12 months	0.764 (0.312-1.869)	0.56		
		Over 12 months	1.00			
	Time treating TB					
		Up to 2 months	1.915 (0.458-8.008)	0.37		
		Over 2 months	1.00			
	Hospitalized on date of interview					
		Yes	0.385 (0.096-1.547)	0.18	0.385 (0.096-1.547)	0.179
		No	1.00		1.00	

95%CI: 95% confidence interval; TB: tuberculosis.

**Table 4:** Factors associated with drug therapy problems - effectiveness

Variable*			Univariate analysis		Multivariate analysis	
			Odds ratio (95%CI)	p value	Odds ratio (95%CI)	p value
Sociodemographic						
	Sex					
		Male	0.0615 (0.064-0.591)	0.02		
		Female	1.00			
	Age					
		>40 years	0.25 (0.267-2.341)	0.23		
		≤40 years	1.00			
	Place of residence					
		Metropolitan region	0.677 (0.069-6.635)	0.74		
		Other municipalities	1.00			
	Marital status					
		Single or widowed	0.178 (0.027-1.156)	0.07	0.074 (0.006-0.878)	0.03
		Married or common law union	1.00		1.00	
	Occupation					
		Yes	1.630 (0.172-15.410)	0.67		
		No	1.00			
Behavioral						
	Smoker					
		No	1.057 (0.167-6.703)	0.953		
		Yes	1.00			
	Alcohol					
		Yes	0.685 (0.108-4.335)	0.69		
		No	1.00			
Clinical						
	Type of TB diagnosis					
		Likely	0.213 (0.022-1.999)	0.18		
		Confirmed	1.00			
	Time since HIV/AIDS diagnosis					
		Up to 12 months	0.524 (0.083-3.319)	0.49		
		Over 12 months	1.00			
	Hospitalized on date of interview					
		Yes	0.677 (0.069-6.635)	0.74		
		No	1,00			
	New case of TB					
		Yes	0.101 (0.014-0.681)	0.02	0.045 (0.004-0.535)	0.01
		No	1.00		1.00	

* The variables ethnicity, time treating tuberculosis, and number of drugs were tested, but presented complete collinearity with the predictor and were not included in the model. 95%CI: confidence interval; TB: tuberculosis.

**Table 5:** Factors associated with drug therapy problems - safety

Variable*			Univariate analysis		Multivariate analysis	
			Odds ratio (95%CI)	p value	Odds ratio (95%CI)	p value
Sociodemographic						
	Sex					
		Male	0.139 (0.012-1.631)	0.12		
		Female	1.00			
	Age					
		>40 years	0.526 (0.046-6.046)	0.61		
		≤40 years	1.00			
	Ethnicity					
		Black or brown	0.091 (0.008-1.083)	0.06	0.039 (0.02-0.777)	0.03
		White	1.00		1.00	
	Occupation					
		Yes	0.786 (0.068-9.11)	0.85		
		No	1.000			
Behavioral						
	Smoker					
		No	1.422 (0.124-16.364)	0.78		
		Yes	1.00			
	Alcohol					
		Yes	0.513 (0.045-5.897)	0.59		
		No	1.00			
Clinical						
	Type of TB diagnosis					
		Likely	1.9 (0.165-21.824)	0.61		
		Confirmed	1.00			
	Time since HIV/AIDS diagnosis					
		Up to 12 months	1.667 (0.145-19.160)	0.68		
		Over 12 months	1.00			
	New case of TB					
		Yes	0.354 (0.030-4.334)	0.42		
		No	1.00			

* The variables place of residence, marital status, time treating tuberculosis, hospitalization on date of interview, and number of drugs presented complete collinearity with the predictor and were not included in the model. 95%CI: confidence interval; TB: tuberculosis.

**Table 6:** Factors associated with drug therapy problems - compliance

Variable			Univariate analysis		Multivariate analysis	
			Odds ratio (95%CI)	p value	Odds ratio (95%CI)	p value
Sociodemographic						
	Sex					
		Male	1.042 (0.372-2.915)	0.94		
		Female	1.00			
	Age					
		>40 Years	0.857 (0.358-2.052)	0.73		
		≤40 Years	1.00			
	Ethnicity					
		Black or brown	1.855 (0.562-6.118)	0.31		
		White	1.00			
	Place of residence					
		Metropolitan region	3.273 (0.816-13.132)	0.09		
		Other municipalities	1.00			
	Marital status					
		Single or widowed	2.466 (0.830-7.321)	0.10	2.96 (0.833-10.48)	0.09
		Married or common law union	1.00		1.00	
	Occupation					
		Yes	0.487 (0.182-1.305)	0.15		
		No	1.00			
Behavioral						
	Smoker					
		No	0.546 (0.222-1.343)	0.19		
		Yes	1.00			
	Alcohol					
		Yes	2.246 (0.918-5.496)	0.08		
		No	1.00			
Clinical						
	Type of diagnosis of TB					
		Likely	0.428 (0.176-1.044)	0.06		
		Confirmed	1.00			
	Time since HIV/AIDS diagnosis					
		Up to 12 months	0.185 (0.071-0.482)	0.001	4.125 (0.734-23.17)	0.11
		Over 12 months	1.00		1.00	
	Time treating TB					
		Up to 2 months	2.844 (0.680-11.895)	0.15	0.222 (0.008-0.637)	0.005
		Over 2 months	1.00		1.00	
	Hospitalization on the date of interview					
		Yes	0.618 (0.179-2.138)	0.45		
		No	1.00			
	New case of TB					
		Yes	0.127 (0.026-0.619)	0.01	0.18 (0.031-1.02)	0.05
		No	1.00		1.00	
	Pharmacotherapeutical					
		Yes	0.618 (0.179-2.138)	0.45		
		No	1.00			

95%CI: 95% confidence interval; TB: tuberculosis.

There was no significant association in the multivariate analysis for indication DTP.

In the final complete model, the variables marital status single and new case were associated with a lower likelihood of effectiveness DTP; white ethnicity, higher likelihood of safety DTP, new case, time since HIV diagnosis less than one year, marital status married or common law union, and time treating TB more than 2 months were more likely to have compliance DTP and remained in the model. The variables marital status and time treating HIV continued in the model, despite p>0.05, because they presented collinearity in the analysis of compliance DTP.

## DISCUSSION

The high frequency of DTP in coinfected patients observed in the present study can be attributed to the severe profile of patients, who had major immunodeficiencies, were on multiple drugs to treat coinfection and other associated conditions, in addition to prophylaxis of opportunistic infections and adverse reactions. Frequently, these patients need hospitalization and to be referred to other health professionals. Previous studies have described DTP or medication errors identified by pharmacists and their intervention for patients with TB^(^^10^^–^^12^^)^ or HIV/AIDS.^(^^13^^–^^15^^)^ As far as we aware of, this is one of the first studies to assess DTP in patients coinfected with TB and HIV/AIDS.

Publications that assess DTP for patients with TB and HIV/AIDS are incipient in Brazil and in the world, and classification methods are different, which makes it difficult to compare studies. There is currently a great diversity of definitions used to refer to patient care and safety. Uniform terminologies are important for physicians and pharmacists to use the same terms to benefit patients.^(^^16^^,^^17^^)^

Pharmacists, as members of the multiprofessional care team of coinfected patients, contribute to the assessment of aspects related to medication dose, drug interactions, omission and inadequate continuity of therapy, optimizing clinical outcomes.^(^^10^^–^^11^^)^

TB and HIV/AIDS coinfection is a worldwide public health problem, and TB, a treatable and curable disease, is the major cause of hospitalization and death among patients with HIV.^(^^18^^)^ TB treatment outcomes for patients with HIV are not satisfactory.^(^^19^^)^ In our study, a considerable number of patients died, abandoned treatment or were transferred even before being recruited to the study, which shows the need to assess pharmacotherapy of coinfected patients and the factors associated with DTP, in addition to driving public policies toward coinfected individuals. In this way, assuring medication that is appropriate for the clinical conditions presented by patients, and that is effective and safe, which patients comply to treatment, can have a major impact on outcomes.

A high frequency of patients with DTP was observed, similarly to previous studies that assessed the impact of drug therapy management on patient care and that used the same classification method for DTP.^(^^20^^–^^22^^)^

Indication DTP informs about an untreated health condition, indicating the need for inclusion of a new drug in a patient's pharmacotherapy. In the present study, most of the DTP found were indication, due to the absence of prescription of pyridoxine. The finding shows the need to train prescribers on prophylaxis of peripheral neuropathy during treatment with isoniazid. Peripheral neuropathy may occur in roughly 20% of cases of TB, and the risk increases in HIV-infected patients.^(^^23^^)^ Studies have suggested that when pharmacists work in collaboration with patients and other healthcare professionals to reach therapeutic goals, the use of medication increases due to clinical conditions not previously identified.^(^^21^^)^ A major part of patients was not on an antiretroviral treatment regimen and, among those who were, most were on the first line treatment recommended in Brazil at the time of the study. Many patients were diagnosed near the time of the interview and were patients with severe disease, hospitalized, with pronounced immunosuppression, indicating a possible late diagnosis of AIDS.

For pharmacotherapy, compliance to treatment is analyzed after checking if medication is indicated, effective and safe. Compliance DTP measures if the patient follows the treatment established by the physician. Despite antiretroviral therapy and anti-TB medication being available in the Unified Health System (SUS – *Sistema* Único *de Saúde*), patients do not comply to therapies, as our study has shown. Causes for non-compliance to pharmacotherapy are multifactorial and may be related to several factors such as the patient, deficiencies in the healthcare system, healthcare team, socioeconomic factors, disease and drug treatment.^(^^24^^)^ When analyzing causes of non-compliance to treatment based on patient reports, we found that most patients did not comply because they preferred not to use medication. It is always important to understand the experience of using medication, as the experience can influence the patient to decrease or increase dose, or make changes to the therapeutic regimen.^(^^21^^)^ Others studies have found that depression, negative feelings and loss of hope may reduce an individual's motivation to treat.^(^^25^^)^ A study by Rodrigues et al., found that the factors related to non-compliance were smoking and alcohol, poor socioeconomic status, adverse reactions, number of pills and lack of motivation.^(^^26^^)^

There was an association between non-compliance and time on TB treatment in our study. Patients with less than 2 months on TB treatment presented more compliance DTP than individuals on the maintenance phase. Normally, when there is symptom remission after the beginning of TB treatment, patients prefer to abandon, believing they are already cured.^(^^27^^)^ Time since HIV diagnosis over 12 months was also associated with non-compliance to treatment. Studies can be associated with the chronic aspect of the disease, leading to psychological stress and disbelief in pharmacotherapy results, leading to treatment abandonment.^(^^27^^)^

There was an association between age and the presence of DTP. As age progresses, more diseases and more medications are observed, mainly in patients within the profile of our study, most of them hospitalized with severe immunodeficiency (T CD4 lymphocytes <200), which may cause the emergence of opportunistic infections, requiring prophylaxis. Polypharmacy associated with age of patients with HIV has already been described in the literature,^(^^28^^)^ and the presence of DTP in patients over 40 years of age was prevalent in our study. Thus, older patients use more medication and have more DTP.

Effectiveness DTP provides information on reach of therapeutic results. Marital status single was associated with higher likelihood of adhering to therapy proposed and less likely to reach objectives proposed. Studies focusing on lack of social support describe that single patients are more vulnerable to non- compliance to therapy, although there are studies reporting that stressor agents, such as affective disorders, may compromise quality of life of individuals and medication use,^(^^29^^)^ which could lead to therapeutic ineffectiveness.

New cases, *i.e*., never having been submitted to anti-TB treatment or on treatment for less than 30 days,^(^^5^^)^ were less likely to reach therapeutic results and compliance to therapy. Thus, patients with a history of abandonment are more likely to interrupt or comply with at unsatisfactory levels,^(^^25^^)^ causing effectiveness DTP.

The directly observed therapy strategy aims to strengthen patient treatment compliance and prevent emergence of strains resistant to TB treatment medication, reducing cases of abandonment and increasing likelihood of cure.^(^^5^^)^ Integrated action between pharmacists and physicians, and the directly observed therapy strategy are important in the care of coinfected patients to increase treatment compliance and effectiveness.

Adverse reactions and medication errors in patient pharmacotherapy are classified as safety DTP. The association between white ethnicity and safety DTP is not consonant with studies showing higher likelihood of adverse reactions for black ethnicity, due to the genetic variability of individuals, which can interfere with enzymes related to the metabolism of pharmaceuticals. However, ethnicity was self-reported by patients.^(^^30^^)^

The present study has limitations. The first is the study being carried out in only one reference hospital for infectious and communicable diseases, which appoints toward caution with generalization of results to all TB and HIV/AIDS coinfected patients in Belo Horizonte (MG), Brazil. Another limiting factor is not having a pharmacotherapeutic follow-up of patients. However, knowing the major DTP presented by these patients and associated factors is very important to define which are the priority patients for pharmacotherapy follow-up.

## CONCLUSION

Tuberculosis and HIV/AIDS coinfected individuals aged over 40 years are more likely to have drug therapy problems during treatment; in that, the most frequent are those that signal toward need of medication for an untreated health condition and non-compliance to treatment. Single patients with a diagnosis of tuberculosis made less than 2 months, HIV/AIDS follow-up time over 12 months and who are not new cases should be a priority for follow-up by a multiprofessional healthcare team to foster adherence. Drug safety related problems point toward a higher likelihood of adverse reactions in white ethnic individuals.

Acknowledging factors associated with the occurrence of drug therapy-related problems in tuberculosis and HIV/AIDS coinfected patients enables the healthcare team to implement measures to guarantee indicated, effective and safe medication for their clinical status and awareness of the need to adhere to the treatment proposed, an important action to improve the results in care of these patients, avoiding therapeutic failure and emergence of multi-resistance.
