# Supplementary material for: Drug therapy problems for patients with tuberculosis and HIV/AIDS at a reference hospital
Source: Einstein (Sao Paulo). 2019 Aug 19;17(4):eAO4696. doi: 10.31744/einstein_journal/2019AO4696 (PMC6706227; doi:10.31744/einstein_journal/2019AO4696)
Supplement: Translation

# Translation: Problemas relacionados ao uso de medicamentos em pacientes com tuberculose e HIV/AIDS em hospital referência

**Authors:** Natália Helena de Resende, Silvana Spíndola de Miranda, Maria das Graças Braga Ceccato, João Paulo Amaral Haddad, Adriano Max Moreira Reis, Dirce Inês da Silva, Wânia da Silva Carvalho

**Affiliations:** 1 Universidade Federal de Minas Gerais, Belo Horizonte, MG, Brasil; 2 Fundação Hospitalar do Estado de Minas Gerais, Hospital Eduardo de Menezes, Belo Horizonte, MG, Brasil

**Keywords:** HIV, Síndrome da imunodeficiência adquirida, Tuberculose, Preparações farmacêuticas

## Abstract

**Objetivo:**

Determinar a frequência de problemas relacionados ao uso de medicamentos no tratamento de pacientes com tuberculose e HIV/AIDS.

**Métodos:**

Os dados foram obtidos por estudo transversal realizado entre setembro de 2015 e dezembro de 2016 em hospital referência em doenças infectocontagiosas de Belo Horizonte (MG), Brasil. As variáveis sociodemográficas, clínicas, comportamentais e farmacoterapêuticas foram avaliadas por questionário semiestruturado. Classificaram-se os problemas relacionados ao uso de medicamento empregando o método *Pharmacotherapy Workup* de atenção farmacêutica. Os fatores associados aos problemas relacionados ao uso de medicamentos de indicação, efetividade, segurança e adesão foram avaliados pela regressão logística múltipla.

**Resultados:**

Foram avaliados 81 pacientes, e 80% apresentaram pelo menos um problema relacionado ao uso de medicamentos, sendo os mais frequentes ligados à problemas relacionados ao uso de medicamentos de indicação e adesão. Os fatores associados aos problemas relacionados ao uso de medicamentos foram idade, estado civil, caso novo, etnia, tempo de diagnóstico do HIV e tempo de tratamento da tuberculose.

**Conclusão:**

A frequência de problemas relacionados ao uso de medicamentos em pacientes coinfectados foi alta, e a identificação dos principais problemas relacionados ao uso de medicamentos e dos fatores associados aos mesmos pode direcionar a equipe multiprofissional de saúde, para garantir o uso dos medicamentos mais indicados, efetivos, seguros e convenientes para a condição clínica dos pacientes. Os indivíduos coinfectados com tuberculose e HIV/AIDS maiores de 40 anos possuem maior chance de apresentarem problemas relacionados ao uso de medicamentos durante o tratamento, sendo os mais frequentes os que indicam a necessidade de medicamento para condição de saúde não tratada e não adesão ao tratamento. Pacientes mais idosos, solteiros ou não, que já trataram a tuberculose antes, com menor tempo de tratamento de tuberculose e maior tempo de diagnóstico de HIV/AIDS devem ter atenção especial no acompanhamento por uma equipe multiprofissional de saúde por indicarem maior chance de apresentar Problemas Relacionados ao uso de Medicamentos de não adesão à terapia.

## INTRODUÇÃO

A infecção pelo vírus da imunodeficiência humana (HIV) representa grande desafio para o controle da tuberculose (TB) em todo o mundo. A maior suscetibilidade de desenvolver TB em pacientes com HIV/AIDS é explicada pela menor resposta imunológica ao *Mycobacterium tuberculosis*. Com isso, ocorre rápida progressão do HIV.^(^^1^^)^

No ano de 2016, estimaram-se 10,6 milhões de casos novos de TB no mundo, e que 10% (1,1 milhão) também eram infectados pelo HIV. A coinfecção TB/ HIV-AIDS é responsável pelo aumento dos índices de mortalidade em todo o mundo. Em 2016, estimaram-se 1,5 milhão de mortes por TB em pacientes HIV negativo e adicional de 374 mil pacientes que foram a óbito devido à coinfecção.^(^^2^^)^

O tratamento conjunto de TB e HIV/AIDS diminui a mortalidade, a recidiva de TB e a transmissão das duas doenças na comunidade.^(^^3^^)^ Para os casos novos de TB, o tratamento se baseia na combinação de quatro fármacos, sendo rifampicina, isoniazida, pirazinamida e etambutol na fase intensiva, de duração de dois meses. Os fármacos rifampicina e isoniazida são utilizados por 4 meses na fase de manutenção.^(^^4^^)^ No Brasil, a terapia inicial da AIDS recomendada no período do estudo era incluir combinações de três antirretrovirais, dois inibidores da transcriptase reversa análogos de nucleosídeos (ITRN) associado ao inibidor da transcriptase reversa não análogo de nucleotídeo (ITRNN). O esquema de primeira linha era composto por tenofovir (TDF), lamivudina (3TC) e efavirenz (EFV). A apresentação desse esquema é em dose fixa combinada dos três fármacos. Recomenda-se a utilização da segunda linha do tratamento em situações em que o uso de EFV e nevirapina esteja impossibilitado; nesse caso, deve-se substitui-lo por um inibidor de protease (IP).^(^^5^^)^

A terapia simultânea das duas infecções, com grande número de medicamentos, predispõe à ocorrência de reações adversas, interações medicamentosas e possibilidade de não garantir boa adesão ao tratamento.^(^^6^^,^^7^^)^ Diante desse contexto, a abordagem multiprofissional é muito importante para esses pacientes, a fim de prevenir, identificar e resolver problemas relacionados ao uso de medicamentos (PRM), ou seja, qualquer evento indesejado vivenciado pelo paciente que envolve o tratamento medicamentoso e interfere no alcance dos objetivos terapêuticos desejados.^(^^8^^)^

## OBJETIVO

Determinar a frequência de problemas relacionados ao uso de medicamentos de indicação, efetividade, segurança e adesão no tratamento de pacientes com tuberculose e HIV/AIDS, bem como avaliar os fatores associados.

## MÉTODOS

Estudo transversal do tipo analítico, realizado entre setembro de 2015 e dezembro de 2016, no Hospital Eduardo de Menezes (HEM) da Fundação Hospitalar do Estado de Minas Gerais (FHEMIG) em Belo Horizonte (MG). Trata-se de hospital referência para TB, HIV/AIDS e outras doenças infectocontagiosas.

Este estudo foi aprovado pelo Comitê de Ética em Pesquisa da Universidade Federal de Minas Gerais, número 3434458, CAAE: 23692713.30000.5149 e da FHEMIG, número 696.759, CAAE: 3692713.2.3001.5124.

Foram incluídos os pacientes com diagnóstico de TB e HIV/AIDS, com início do tratamento da TB a partir de setembro de 2015 e concomitantemente com o diagnóstico de HIV/AIDS e com idade igual ou superior a 18 anos.

Foram excluídos os pacientes coinfectados com TB e HIV/AIDS que apresentavam *deficit* de cognição ou anteriormente a abordagem: abandono de tratamento, óbito, transferência para outro serviço de saúde e mudança de diagnóstico.

O cálculo do tamanho amostral foi realizado considerando o erro amostral de 10%, o nível de confiança de 95%, a frequência de 50% de PRM e a população de coinfectados atendidos no hospital em 2014, que foi de 136 pacientes. Dessa forma, obteve-se amostra de 57 e, considerando provável taxa de recusa de 30%, a amostra mínima calculada foi de 74 pacientes.

Os pacientes foram identificados por avaliação do uso de medicamentos anti-TB nas prescrições e nas planilhas de dispensação de medicamentos do serviço de farmácia da instituição investigada. Depois, era verificado o diagnóstico do HIV, em consulta aos prontuários médicos, ao Sistema Integrado de Gestão Hospitalar (SIGH) da FHEMIG) e ao Sistema Logístico de Medicamentos (SICLOM) do Ministério da Saúde do Programa Infecções Sexualmente Transmissíveis/AIDS e Hepatites Virais (http://azt.aids.gov.br/). Os dados foram coletados por entrevistas dos pacientes utilizando questionário semiestruturado no ambulatório, enfermaria e hospital-dia do HEM.

As variáveis explicativas investigadas foram agrupadas em características sociodemográficas e econômicas (sexo, idade, morar sozinho, local de residência, estado civil, escolaridade, etnia, renda e ocupação); características comportamentais (uso de tabaco, álcool e drogas ilícitas); características clínicas (forma clínica da TB, tempo de diagnóstico HIV/AIDS, tempo de tratamento da TB, internação na data da entrevista, caso novo, doenças associadas, carga viral e linfócitos T CD4); e características relacionadas ao tratamento medicamentoso (esquemas de tratamento para TB e HIV/AIDS, número de fármacos utilizados).

As variáveis respostas foram os PRM, que foram investigados por meio do questionário adaptado de Cipolle et al., e classificados pelo método *Pharmacotherapy Workup* de atenção farmacêutica, descrito na tabela 1.^(^^9^^)^

**Tabela 1:** Parâmetros para classificação de cada problema relacionado ao uso de medicamentos

Parâmetro	Classificação de PRM
Necessidade	PRM 1 – Medicamento desnecessário
	A - não há indicação
	B - terapia dupla
	C - terapia não farmacológica indicada
	D - Reação adversa a medicamento previsível e previnível
	E - uso recreativo de fármacos
	PRM 2 – Necessita Farmacoterapia adicional
	A - condição não tratada
	B - preventiva – profilática
	C - sinergismo – potenciação
Efetividade	PRM 3 – Necessita medicamento diferente
	A - medicamento mais efetivo disponível
	B - condição refratária ao medicamento
	C - forma farmacêutica inapropriada
	D - não efetivo para a condição
	PRM 4 – Dose muito baixa
	A - dose errada
	B - frequência inapropriada
	C - interação medicamentosa
	D - duração inapropriada
Segurança	PRM 5 – Reação adversa a medicamento
	A - efeito indesejável
	B - medicamento inseguro para o paciente
	C - administração muito rápida
	D - reação alérgica
	E - contra-indicação presente
	PRM 6 – Dose muito alta
	A - dose incorreta
	B - frequência inapropriada
	C - duração inapropriada
	D - interação medicamentosa
	E - administração incorreta
Cumprimento	PRM 7 – Não segue as instruções
	A - não entende as instruções
	B - paciente prefere não tomar
	C - paciente esquece de tomar
	D - produto muito caro
	E - não consegue engolir ou administrar
	F - produtos não disponível

Fonte: Adaptado de Cipolle RJ, Strand LM, Morley PC. Pharmaceutical care practice: the clinician's guide. 2nd ed. New York: McGraw-Hill; 2004. 394 p.

PRM: problemas relacionado ao uso de medicamentos.

### Análise estatística

Inicialmente, foi realizada análise descritiva dos dados obtidos, que incluiu a descrição da população estudada, e a distribuições de frequências das variáveis categóricas e medidas de tendência central, como média, mediana e desvio padrão, para as variáveis quantitativas.

A análise descritiva incluiu a determinação da proporção de PRM de acordo com as classificações preconizadas.

A associação entre todas as características selecionadas (sociodemográficas, comportamentais, dados clínicos e perfil farmacoterapêutico) e os PRM de indicação, efetividade, segurança e adesão foi avaliada por regressão logística múltipla.

As variáveis independentes com p<0,20 foram incluídas no modelo multivariado. Foi realizado o teste de ajuste do modelo de Hosmer-Lemeshow. Os dados foram analisados utilizando-se o programa Stata versão 12.0 (Stata Corporation, College Station, Estados Unidos).

No modelo final, permaneceram as variáveis que mantiveram p<0,05.

## RESULTADOS

Dos 140 pacientes coinfectados no período do estudo, foram excluídos 59 pacientes, por diferentes motivos (Figura 1). A amostra final para a análise foi composta por 81 pacientes.

**Figura 1:** População do estudo de pacientes coinfectados com tuberculose e HIV/AIDS para inclusão no estudo

Observa-se, na tabela 2, que 62 pacientes (77%) eram do sexo masculino e a média de idade era de 40 anos. Entre os 81 pacientes coinfectados, 63 eram solteiros (78%), 67 se declararam pardos ou negros (83%) e 59 tinham até 8 anos de estudo (73%). A maioria tinha renda (86%) e ocupação (72%).

**Tabela 2:** Características sociodemográficas, comportamentas, clínicas e farmacoterapêutica dos pacientes coinfectados com tuberculose e HIV/AIDS

Variáveis		n (%)
Sociodemográficas		
	Masculino	62 (77)
	>40 anos	39 (52)
	Solteiro	63 (78)
	Até 8 anos de escolaridade	59 (73)
	Pele negra ou parda	67 (83)
	Renda	70 (86)
Comportamentais		
	Tabaco	33 (41)
	Alcoolismo	39 (48)
	Drogas	18 (22)
Clínicas		
	TB pulmonar	46 (57)
	TB extrapulmonar	27 (33)
	TB pulmonar + extrapulmonar	8 (10)
	Doenças associadas	62 (77)
	Diagnóstico do HIV até 1 ano	44 (54)
	Tratamento da TB até 2 meses	71 (88)
	Carga viral indetectável	10 (12)
	Linfócitos T CD4 <200 células/µL	59 (73)
Farmacoterapêuticas		
	Esquema básico de tratamento TB	69 (85)
	Nenhum esquema TARV	40 (49)
	TARV primeira linha	35 (43)
	TARV segunda linha	6 (7)
Número de fármacos		
	<10	12 (15)
	≥10	69 (85)

TB: tuberculose; TARV: terapia antirretroviral.

A forma clínica da TB mais frequente foi a pulmonar (57%), seguida da extrapulmonar (33%). Das apresentações clínicas extrapulmonares, a TB meningoencefálica foi a mais frequente, representando 33% dos pacientes, seguida da ganglionar (26%) e miliar (26%). Dos 81 pacientes, 62 (77%) apresentaram outras doenças associadas, além da coinfecção, como candidíase (23%), pneumonia (12%) e infecção por citomegalovírus (10%). Além disso, observou-se a presença de alcoolismo em 39 pacientes (48%), tabagismo em 33 (41%) e uso de drogas ilícitas em 18 (22%). Das variáveis relacionadas ao tratamento medicamentoso, verificou-se que 69 utilizaram o esquema básico composto por rifampicina, isoniazida, pirazinamida e etambutol para TB (85%) e 40 (49%) não utilizaram qualquer esquema antirretroviral.

Alguns pacientes utilizavam o esquema especial para tratar a TB devido a reações adversas que já estavam sendo tratadas no momento da coleta de dados.

No momento da entrevista, 71 pacientes (88%) estavam tratando a TB por até 2 meses. O diagnóstico do HIV ocorreu em até 1 ano antes da data da entrevista em 44 pacientes (54%). A maioria dos pacientes sem terapia antirretroviral estava na fase intensiva do tratamento da TB (95%).

Dos 81 pacientes entrevistados, 65 (80,0%) tinham pelo menos um PRM, e 46 pacientes (57%) apresentaram PRM de necessidade, 39 (48%) de adesão ao tratamento, 5 (6%) de efetividade e 3 (4%) de segurança.

Foram identificados 110 PRM, dos quais 57 (52%) eram relacionados à necessidade de farmacoterapia adicional. Desses, 20 (35%) eram devido a condições não tratadas e 37 (65%) à necessidade de profilaxia. A necessidade de suplementação da piridoxina devido ao risco de neuropatia periférica foi classificada como PRM de necessidade de profilaxia.

Observou-se 39 PRM de não adesão ao tratamento (35%). As causas para a não adesão foram: preferir não tomar os medicamentos (53,8%), esquecer de tomar os medicamentos (33%), medicamento não disponível (8%) e não entender as instruções (5%).

Na análise univariada das variáveis categóricas, segundo a presença ou ausência de qualquer tipo de PRM, a única variável que apresentou significância foi a idade (valor p=0,046), a chance foi 3,5 vezes maior de apresentar PRM para indivíduos com mais de 40 anos (intervalo de confiança de 95% − IC95%: 1,020-12,000).

Nas tabelas 3 a 6 estão apresentados os resultados da análise univariada e multivariada com as características associadas aos PRM de indicação, efetividade, segurança e adesão, respectivamente.

**Tabela 3:** Fatores associados a problemas relacionados ao uso de medicamentos - indicação

Variável			Análise univariada		Análise multivariada	
			*Odds ratio* (IC95%)	Valor de p	*Odds ratio* (IC95%)	Valor de p
Sociodemográficas						
	Sexo					
		Masculino	1,246 (0,444-3,498)	0,676		
		Feminino	1,00			
	Idade					
		>40 anos	1,786 (0,733-4,353)	0,20		
		≤40 anos	1,00			
	Etnia					
		Negra ou parda	0,465 (0,132-1,630)	0,23		
		Branca	1,00			
	Local de residência					
		Região metropolitana	1,379 (0,404-0,711)	0,61		
		Outros municípios	1,00			
	Estado civil					
		Solteiro ou viúvo	0,525 (0,177-1,560)	0,25		
		Casado ou união				
	Ocupação					
		Sim	0,612 (0,225-1,667)	0,34		
		Não	1,00			
Comportamentais						
	Tabaco					
		Não	0,995 (0,404-2,448)	0,99		
		Sim	1,00			
	Álcool					
		Sim	0,607 (0,249-1,484)	0,27		
		Não	1,00			
Clínicas						
	Tipo de diagnóstico da TB					
		Probabilidade	0,690 (0,284-1,665)	0,41		
		Confirmado	1,00			
	Tempo de diagnóstico HIV/AIDS					
		Até 12 meses	0,764 (0,312-1,869)	0,56		
		Acima de 12 meses	1,00			
	Tempo de tratamento da TB					
		Até 2 meses	1,915 (0,458-8,008)	0,37		
		Mais de 2 meses	1,00			
	Internação na data da entrevista					
		Sim	0,385 (0,096-1,547)	0,18	0,385 (0,096-1,547)	0,179
		Não	1,00		1,00	

IC95%: intervalo de confiança; TB: tuberculose.

**Tabela 4:** Fatores associados a problemas relacionados ao uso de medicamentos - efetividade

Variável*			Análise univariada		Análise multivariada	
			*Odds ratio* (IC95%)	Valor de p	*Odds ratio* (IC95%)	Valor de p
Sociodemográficas						
	Sexo					
		Masculino	0,0615 (0,064-0,591)	0,02		
		Feminino	1,00			
	Idade					
		>40 anos	0,25 (0,267-2,341)	0,23		
		≤40 anos	1,00			
	Local de residência					
		Região metropolitana	0,677 (0,069-6,635)	0,74		
		Outros municípios	1,00			
	Estado civil					
		Solteiro ou viúvo	0,178 (0,027-1,156)	0,07	0,074 (0,006-0,878)	0,03
		Casado ou união	1,00		1,00	
	Ocupação					
		Sim	1,630 (0,172-15,410)	0,67		
		Não	1,00			
Comportamentais						
	Tabaco					
		Não	1,057 (0,167-6,703)	0,953		
		Sim	1,00			
	Álcool					
		Sim	0,685 (0,108-4,335)	0,69		
		Não	1,00			
Clínicas						
	Tipo de tratamento da TB					
		Probabilidade	0,213 (0,022-1,999)	0,18		
		Confirmado	1,00			
	Tempo de diagnóstico HIV/AIDS					
		Até 12 meses	0,524 (0,083-3,319)	0,49		
		Acima de 12 meses	1,00			
	Internação na data da entrevista					
		Sim	0,677 (0,069-6,635)	0,74		
		Não	1,00			
	Caso novo para TB					
		Sim	0,101 (0,014-0,681)	0,02	0,045 (0,004-0,535)	0,01
		Não	1,00		1,00	

* As variáveis etnia, tempo de tratamento da tuberculose e número de fármacos foram testadas, mas apresentaram completa colinearidade com o preditor e não foram incluídas no modelo. IC95%: intervalo de confiança; TB: tuberculose.

**Tabela 5:** Fatores associados a problemas relacionados ao uso de medicamentos - segurança

Variável*			Análise univariada		Análise multivariada*	
			*Odds ratio* (IC95%)	Valor de p	*Odds ratio* (IC95%)	Valor de p
Sociodemográficas						
	Sexo					
		Masculino	0,139 (0,012-1,631)	0,12		
		Feminino	1,00			
	Idade					
		>40 anos	0,526 (0,046-6,046)	0,61		
		≤40 anos	1,00			
	Etnia					
		Negra ou parda	0,091 (0,008-1,083)	0,06	0,039 (0,02-0,777)	0,03
		Branca	1,00		1,00	
	Ocupação					
		Sim	0,786 (0,068-9,11)	0,85		
		Não	1,00			
Comportamentais						
	Tabaco					
		Não	1,422 (0,124-16,364)	0,78		
		Sim	1,00			
	Álcool					
		Sim	0,513 (0,045-5,897)	0,59		
		Não	1,00			
Clínicas						
	Tipo de diagnóstico da TB					
		Probabilidade	1,9 (0,165-21,824)	0,61		
		Confirmado	1,00			
	Tempo de diagnóstico HIV/AIDS					
		Até 12 meses	1,667 (0,145-19,160)	0,68		
		Acima de 12 meses	1,00			
	Caso novo para TB					
		Sim	0,354 (0,030-4,334)	0,42		
		Não	1,00			

* As variáveis local de residência, estado civil, tempo de tratamento da tuberculose, internação na data da entrevista, e quantidade de fármacos apresentaram completa colinearidade com o preditor e não foram incluídas no modelo. IC95%: intervalo de confiança; TB: tuberculose.

**Tabela 6:** Fatores associados a problemas relacionados ao uso de medicamentos - adesão

Variável			Análise univariada		Análise multivariada	
			*Odds ratio* (IC95%)	Valor de p	*Odds ratio* (IC95%)	Valor de p
Sociodemográficas						
	Sexo					
		Masculino	1,042 (0,372-2,915)	0,94		
		Feminino	1,00			
	Idade					
		>40 anos	0,857 (0,358-2,052)	0,73		
		≤40 anos	1,00			
	Etnia					
		Negra ou parda	1,855 (0,562-6,118)	0,31		
		Branca	1,00			
	Local de residência					
		Região metropolitana	3,273 (0,816-13,132)	0,09		
		Outros municípios	1,00			
	Estado civil					
		Solteiro ou viúvo	2,466 (0,830-7,321)	0,10	2,96 (0,833-10,48)	0,09
		Casado ou união	1,00		1,00	
	Ocupação					
		Sim	0,487 (0,182-1,305)	0,15		
		Não	1,00			
Comportamentais						
	Tabaco					
		Não	0,546 (0,222-1,343)	0,19		
		Sim	1,00			
	Álcool					
		Sim	2,246 (0,918-5,496)	0,08		
		Não	1,00			
Clínicas						
	Tipo de diagnóstico da TB					
		Probabilidade	0,428 (0,176-1,044)	0,06		
		Confirmado	1,00			
	Tempo de diagnóstico HIV/AIDS					
		Até 12 meses	0,185 (0,071-0,482)	0,001	4,125 (0,734-23,17)	0,11
		Acima de 12 meses	1,00		1,00	
	Tempo de tratamento da TB					
		Até 2 meses	2,844 (0,680-11,895)	0,15	0,222 (0,008-0,637)	0,005
		Mais de 2 meses	1,00		1,00	
	Internação na data da entrevista					
		Sim	0,618 (0,179-2,138)	0,45		
		Não	1,00			
	Caso novo para TB					
		Sim	0,127 (0,026-0,619)	0,01	0,18 (0,031-1,02)	0,05
		Não	1,00		1,00	
	Farmacoterapêuticas					
		Sim	0,618 (0,179-2,138)	0,45		
		Não	1,00			

IC95%: intervalo de confiança; TB: tuberculose.

Não houve associação significativa na análise multivariada dos PRM de indicação.

No modelo final completo, as variáveis estado civil solteiro e caso novo estavam associadas a menor chance de PRM de efetividade; etnia branca, maior chance de PRM de segurança, caso novo, tempo de diagnóstico do HIV menor que 1 ano, estado civil casado ou união, e tempo de tratamento de TB mais de 2 meses tiveram menor chance de PRM de adesão e permaneceram no modelo. As variáveis estado civil e tempo do tratamento do HIV continuaram no modelo, apesar do valor p>0,05, porque apresentaram colinearidade na análise com os PRM de adesão.

## DISCUSSÃO

A alta frequência de PRM em pacientes coinfectados detectada neste estudo pode ser atribuída ao perfil de gravidade dos pacientes, que apresentam imunodeficiência importante, utilizam múltiplos medicamentos para tratamento da coinfecção e outras doenças associadas, além de profilaxias de infecções oportunistas e reações adversas. Frequentemente, esses pacientes necessitam de internação e encaminhamento a outros profissionais de saúde. Estudos prévios descreveram PRM ou erros de medicação identificados por farmacêuticos e suas intervenções em pacientes com TB^(^^10^^–^^12^^)^ ou HIV/AIDS.^(^^13^^–^^15^^)^ Do nosso conhecimento, este é um dos primeiros estudos a avaliar PRM em pacientes coinfectados com TB e HIV/AIDS.

As publicações que avaliam PRM em pacientes com TB e HIV/AIDS são incipientes no Brasil e no mundo, e as metodologias de classificação são distintas, o que dificulta a comparabilidade dos estudos. Atualmente, existe grande diversidade nas definições utilizadas no que se refere ao cuidado e à segurança do paciente. A uniformização das terminologias é importante a fim de que médicos e farmacêuticos utilizem os mesmos termos para o benefício do paciente.^(^^16^^,^^17^^)^

Os farmacêuticos, como membros da equipe multiprofissional de cuidado aos pacientes coinfectados, contribuem para a avaliação dos aspectos relacionados à dose dos medicamentos, interações medicamentosas, omissão e continuidade inadequada da terapia, otimizando os resultados clínicos.^(^^10^^–^^11^^)^

A coinfecção TB e HIV/AIDS é um problema de saúde pública no mundo, e a TB, doença tratável e curável, é a maior causa de hospitalização e mortes entre os pacientes com HIV.^(^^18^^)^ Os desfechos do tratamento da TB em pacientes com HIV não são satisfatórios.^(^^19^^)^ Em nosso estudo, grande parte dos pacientes foram a óbito, abandonaram o tratamento ou foram transferidos antes mesmo da abordagem para a participação da pesquisa, o que mostra a necessidade de avaliar a farmacoterapia de pacientes coinfectados e os fatores associados aos PRM, além de direcionar políticas públicas para os coinfectados. Dessa forma, garantir que os medicamentos sejam apropriados para as condições clínicas apresentadas pelos pacientes, que estes sejam efetivos, seguros, e que haja adesão aos tratamentos poderá ter grande impacto nos desfechos.

Observou-se alta frequência de pacientes com PRM, assim como em outros estudos que avaliaram o impacto do gerenciamento da terapia medicamentosa no cuidado a pacientes e que utilizaram a mesma metodologia de classificação de PRM.^(^^20^^–^^22^^)^

O PRM de indicação informa sobre uma condição de saúde não tratada, indicando a necessidade de inclusão de um novo medicamento na farmacoterapia do paciente. No presente estudo, a maioria dos PRM encontrados eram de indicação, devido à ausência da prescrição de piridoxina. Isso demonstra a necessidade de orientar os prescritores sobre a profilaxia de neuropatia periférica durante o tratamento com isoniazida. A neuropatia periférica pode ocorrer em cerca de 20% dos casos de TB, e esse risco aumenta em pacientes infectados pelo HIV.^(^^23^^)^ Estudos sugerem que quando farmacêuticos atuam em colaboração com os pacientes e outros profissionais de saúde para o alcance dos objetivos terapêuticos, aumenta-se a utilização de medicamentos devido a condições clínicas que não foram identificadas previamente.^(^^21^^)^ Em relação ao uso da terapia antirretroviral, grande parte dos pacientes estava sem qualquer esquema terapêutico e, entre os que estavam, a maioria usava o tratamento de primeira linha preconizado no Brasil no momento do estudo. Muitos pacientes receberam o diagnóstico próximo da entrevista e eram pacientes graves, internados, com imunossupressão acentuada, indicando possível diagnóstico tardio da AIDS.

Na avaliação farmacoterapêutica, após verificar se o medicamento é indicado, efetivo e seguro, analisa-se a adesão ao tratamento. O PRM de adesão mensura se o paciente segue o tratamento estabelecido com o médico. Apesar de a terapia antirretroviral e os medicamentos anti-TB estarem disponíveis no Sistema Único de Saúde (SUS), os pacientes não aderem às terapias, como mostrado em nosso estudo. As causas para a não adesão à farmacoterapia são multifatoriais e podem estar relacionadas a diversos fatores, como o paciente, a deficiência no sistema de saúde, a equipe de saúde, os fatores socioeconômicos, a doença e o tratamento medicamentoso.^(^^24^^)^ Quando se avaliaram as causas da não adesão ao tratamento de acordo com o relato do paciente, observou-se que a maioria dos pacientes não aderia por preferir não utilizar os medicamentos. É sempre importante entender a experiência com o uso de medicamentos, pois essa pode influenciar o paciente a diminuir ou aumentar a dose, ou a fazer modificações em seu regime terapêutico.^(^^21^^)^ Outros estudos verificaram que depressão, sentimentos negativos e perda de esperança podem reduzir a motivação do indivíduo em tratar.^(^^25^^)^ No estudo de Rodrigues et al., os fatores relacionados à não adesão eram uso de tabaco e álcool, baixas condições socioeconômicas, reações adversas, quantidade de comprimidos e falta de motivação.^(^^26^^)^

Em nosso estudo, houve associação da não adesão com o tempo de tratamento da TB. Os pacientes com menos de 2 meses de tratamento da TB têm mais PRM de adesão quando comparados àqueles que estão na fase de manutenção. Normalmente, com a ocorrência da remissão dos sintomas após o início do tratamento da TB os pacientes preferem abandonar, com a crença de que já estão curados.^(^^27^^)^ O tempo de diagnóstico do HIV maior que 12 meses também teve associação com a não adesão ao tratamento. Estudos demonstram que essa dificuldade de adesão pode estar associada ao caráter crônico da doença, causando estresse psicológico e descrença nos resultados da farmacoterapia, levando ao abandono.^(^^27^^)^

Houve associação da idade com a presença de PRM. Com o aumento da idade, observa-se a presença de mais doenças e utilização de grande número de medicamentos, principalmente em pacientes com o perfil do nosso estudo, a maioria internados, com imunodeficiência grave (linfócitos T CD4<200), que pode ocasionar o aparecimento de infecções oportunistas, com necessidade de profilaxias. A polifarmácia associada à idade em pacientes com HIV já é descrita na literatura,^(^^28^^)^ e a presença de PRM em pacientes com idade maior que 40 anos foi prevalente em nosso trabalho. Dessa forma, pacientes que são mais idosos utilizam mais medicamentos e apresentam mais PRM.

O PRM de efetividade informa sobre o alcance dos resultados terapêuticos. O estado civil solteiro esteve associado com maior chance de aderir a terapia proposta e menor chance para alcançar os objetivos propostos. Em estudos que abordam a falta de suporte social, é descrito que pacientes solteiros são mais vulneráveis a não aderirem à terapia, entretanto existem estudos que relatam que agentes estressores, como problemas afetivos, podem comprometer a qualidade de vida dos indivíduos e a utilização dos medicamentos,^(^^29^^)^ o que poderia levar à inefetividade terapêutica.

O fato de o paciente ser caso novo, ou seja, nunca ter se submetido ao tratamento anti-TB ou ter realizado por menos de 30 dias,^(^^5^^)^ teve menor chance para alcançar os resultados terapêuticos e aderir à terapia. Dessa forma, pacientes com histórico de abandono têm maior probabilidade de interromper ou aderir em níveis insatisfatórios,^(^^25^^)^ causando PRM de efetividade.

A estratégia do tratamento diretamente observado objetiva o fortalecimento da adesão do paciente ao tratamento e a prevenção do aparecimento de cepas resistentes aos medicamentos para tratar a TB, reduzindo casos de abandono e aumentando a probabilidade de cura.^(^^5^^)^ Ações integradas do farmacêutico com o médico, assim como a estratégia tratamento diretamente observada, são importantes no cuidado aos pacientes coinfectados para aumentar a adesão e a efetividade do tratamento.

A presença de reações adversas e erros de medicação na farmacoterapia do paciente é sistematizada na categoria dos PRM de segurança. A associação entre a etnia branca e PRM de segurança não está consonante estudos que mostram maior probabilidade de reações adversas em etnia negra, devido à variabilidade genética dos indivíduos, que pode interferir em enzimas relacionadas ao metabolismo de fármacos. Entretanto, deve-se considerar que a etnia foi autorrelatada pelos pacientes.^(^^30^^)^

O presente estudo apresenta limitações. A primeira é a realização do estudo em apenas um hospital de referência para doenças infectocontagiosas, o que indica a necessidade de cautela com a generalização dos resultados para todos os pacientes coinfectados com TB e HIV/AIDS em Belo Horizonte (MG), Brasil. Outro fator limitante é não ter realizado o seguimento farmacoterapêutico dos pacientes. No entanto, conhecer quais são os principais PRM apresentados por esses pacientes e os fatores associados é muito importante para definir quais os pacientes prioritários no acompanhamento da farmacoterapia.

## CONCLUSÃO

Os indivíduos coinfectados com tuberculose e HIV/AIDS maiores de 40 anos possuem maior chance de apresentarem problemas relacionados ao uso de medicamentos durante o tratamento, sendo os mais frequentes os que indicam a necessidade de medicamento para condição de saúde não tratada e não adesão ao tratamento. Pacientes solteiros com diagnóstico de tuberculose há menos de 2 meses, tempo de acompanhamento de HIV/AIDS maior que 12 meses e que não são casos novos devem prioritariamente ser selecionados para acompanhamento por equipe multiprofissional de saúde para a promoção da adesão. Os problemas relacionados ao uso de medicamentos de segurança indicam maior chance de ocorrer reações adversas em indivíduos da etnia branca.

Conhecer os fatores associados à ocorrência de problemas relacionados ao uso de medicamentos em pacientes coinfectados com tuberculose e HIV/AIDS propicia que a equipe de saúde implemente medidas para garantir os medicamentos indicados, efetivos e seguros para sua condição clínica e a consciência da necessidade de aderir ao tratamento proposto, ação importante para melhorar os resultados nos cuidados desses pacientes, evitando falha terapêutica e aparecimento de multirresistência.
